# Manipulating RKIP reverses the metastatic potential of breast cancer cells

**DOI:** 10.3389/fonc.2023.1189350

**Published:** 2023-07-04

**Authors:** Trang Huyen Lai, Mahmoud Ahmed, Jin Seok Hwang, Md Entaz Bahar, Trang Minh Pham, Jinsung Yang, Wanil Kim, Rizi Firman Maulidi, Dong-Kun Lee, Dong-Hee Kim, Hyun Joon Kim, Deok Ryong Kim

**Affiliations:** ^1^Department of Biochemistry and Convergence Medical Sciences and Institute of Health Sciences, Gyeongsang National University College of Medicine, Jinju, Republic of Korea; ^2^Department of Physiology and Convergence Medical Sciences and Institute of Health Sciences, Gyeongsang National University College of Medicine, Jinju, Republic of Korea; ^3^Department of Orthopaedic Surgery and Institute of Health Sciences, Gyeongsang National University Hospital, and Gyeongsang National University College of Medicine, Jinju, Republic of Korea; ^4^Department of Anatomy and Convergence Medical Sciences and Institute of Health Sciences, Gyeongsang National University College of Medicine, Jinju, Republic of Korea

**Keywords:** breast cancer, metastasis, RKIP, SNAI1, NME1, MDA-MB-231, MCF-7

## Abstract

Breast cancer is a common tumor type among women, with a high fatality due to metastasis. Metastasis suppressors encode proteins that inhibit the metastatic cascade independent of the primary tumor growth. Raf kinase inhibitory protein (RKIP) is one of the promising metastasis suppressor candidates. RKIP is reduced or lost in aggressive variants of different types of cancer. A few pre-clinical or clinical studies have capitalized on this protein as a possible therapeutic target. In this article, we employed two breast cancer cells to highlight the role of RKIP as an antimetastatic gene. One is the low metastatic MCF-7 with high RKIP expression, and the other is MDA-MB-231 highly metastatic cell with low RKIP expression. We used high-throughput data to explore how RKIP is lost in human tissues and its effect on cell mobility. Based on our previous work recapitulating the links between RKIP and SNAI, we experimentally manipulated RKIP in the cell models through its novel upstream NME1 and investigated the subsequent genotypic and phenotypic changes. We also demonstrated that RKIP explained the uneven migration abilities of the two cell types. Furthermore, we identified the regulatory circuit that might carry the effect of an existing drug, Epirubicin, on activating gene transcription. In conclusion, we propose and test a potential strategy to reverse the metastatic capability of breast cancer cells by chemically manipulating RKIP expression.

## 1 Introduction

Breast cancer is the second most common disease among American women, following skin cancer, according to the Centers for disease control and Prevention (CDC) (1). Metastasis causes the great majority of fatalities in the final stage of the disease (2). Cancer metastasis is a complex process of disseminating cancer cells from the primary solid tumors to distant parts of the body where they form secondary growth sites (3). Advances in understanding the underlying molecular machinery and mechanisms of metastatic growth have led to improvements in clinical staging and patient survival rates.

Metastasis suppressor genes (MSGs) encode proteins that inhibit various steps in the metastatic cascade independent of the primary tumor growth (4). By contrast, tumor suppressor genes suppress primary tumor growth. Studying MSGs provides insight into the molecular mechanisms that govern metastatic spread and growth in secondary tumor sites. Raf kinase inhibitory protein (RKIP), also known as PEBP1, is a promising metastasis suppressor candidate (5). Functionally, RKIP is a negative regulator of the RAF/MEK/ERK cascade, a crucial pathway connecting signals from cell surface receptors to transcription factors (6). Many works in cell culture and animal models have demonstrated a role for RKIP in preventing the metastatic spread of various cancers, including prostate cancer, breast cancer, and melanoma (7, 8). RKIP was commonly found to be reduced or absent in metastatic variants of established cell lines derived from these cancers. Additionally, RKIP induction has been shown to inhibit the EMT-related gene products such as Notch1 intracellular domain (NICD), Vimentin, and SNAI1 while upregulating the epithelial E-Cadherin (9, 10).

Although extensively studied, few pre-clinical or clinical studies have capitalized on RKIP as a possible therapeutic agent. The lack of application indicates the need for a deeper understanding of the function of RKIP as an anti-metastatic gene and the possible targeting strategies. Here, we use two cell lines to model the migratory patterns of breast cancer cells and their association with the intrinsic levels of RKIP. Furthermore, we build on prior knowledge and previous work from our laboratory to identify the regulators of RKIP. We verified the predicted effects of existing drugs on RKIP expression. Finally, we investigate the transcriptional activity of the gene and the consequent change in cell phenotypes upon treatment with one of these cancer drugs.

## 2 Results

### 2.1 Receptor status, but not copy number, mediates RKIP loss in breast cancer tissues

Loss of RKIP has long been recognized to associate with the more aggressive forms of many tumors, including breast cancer (7). Using gene expression data from human breast tissues, we sought to explain the ways by which RKIP is lost or downregulated. We compared the expression of RKIP in two cohorts of patients: metastatic breast cancer project (MBC; N = 237) sampled from distant metastatic sites or tumors that had metastasized, and molecular taxonomy of breast cancer international consortium (METABRIC; N = 2509) from primary sites (11, 12). We stratified the samples by known copy number alterations (CNA) and receptor status to test their relationship with the expression of RKIP.

**Figure 1:**
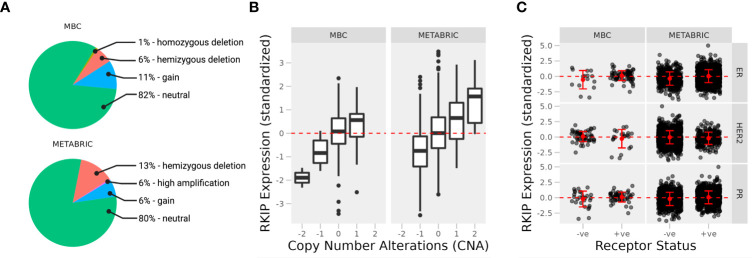
Expression of RKIP in primary and metastatic breast cancer tissues. **(A)** Percentage of cases with copy number variations (CNA) in two cohorts of breast cancer patients; Metastatic breast cancer (MBC; N = 237; primary and metastatic samples) and Molecular Taxonomy of Breast Cancer International Consortium (METABRIC; N = 2509; primary samples). The expression of the RKIP gene was stratified by CNA **(B)** and receptor status **(C)** (+ve, positive; or -ne, negative).

We observed no difference in the overall frequency of CNA between the two cohorts. Only a small percentage of samples were altered or mutated, and most were neutral to copy numbers of the RKIP gene (**Figure 1A**). This suggests that transcriptional or post-transcriptional mechanisms are mainly responsible for the loss of RKIP. Deep gene deletion occurred only in the metastatic tissues, albeit at a low frequency, while gene amplification occasionally appeared in the primary tissues. In the fraction of altered samples, the expression of RKIP correlated strongly with CNA (**Figure 1B**). Furthermore, we observed no correlation between RKIP expression and the hormone receptor status in each sample, except for the estrogen receptor (ER) in the metastatic samples (**Figure 1C**). Metastatic ER-negative samples had lower RKIP expression compared to the positive samples. Together, CNA alterations could explain the changes in RKIP expression but only in a small fraction of samples. Furthermore, the loss of the estrogen receptor correlated with the loss of RKIP in metastatic tissues.

### 2.2 RKIP levels correlate with the migration ability of two breast cancer cell models

MCF-7 is a form of breast adenocarcinoma that proliferate in the presence of estrogen, while MDA-MB-231 is a highly metastatic triple-negative breast cancer (TNBC) cell (13, 14). exploited the uneven ability of the two cell lines to study the effect of RKIP loss on cancer cell migration using scratch assay (**Figure S1**). MCF-7 cells slowly migrated, only occupying 40% of the migration area at 48 h (**Figure S1A** -*upper panel*). Meanwhile, MDA-MB-231 cells exhibited robust, aggressive motility, populating 50% of the migration area at 16h and almost 100% at 48 h (**Figure S1B** - *upper panel*). Our result corroborated previous studies where MCF-7 cells displayed a low metastatic capacity comparedto MDA-MB-231 cells (15).

**Figure 2:**
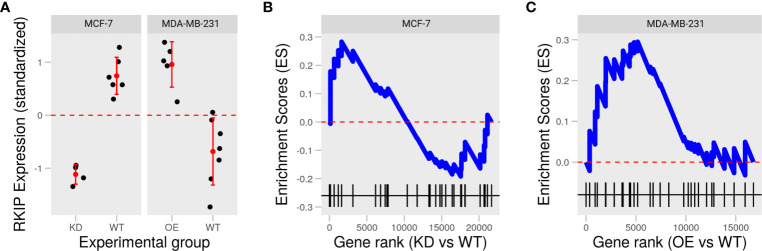
Knockdown and overexpression of RKIP in low and high metastatic breast cancer cells. **(A)** Expression of RKIP in knockdown (KD) or overexpression (OE) compared to the wild-type (WT) MCF-7 and MDA-MB-231 cells in two publically available datasets. **(B, C)** Enrichment scores of the negative regulation of the epithelial to mesenchymal transition (EMT) gene set based on the gene ranks (fold-change) between KD and WT MCF-7 **(B)** and OE and WT MDA-MB-231 **(C)**.

To examine the role of RKIP in two widely studied human breast cancer cell lines: MCF-7 and MDA-MB-231, we collected two publically available datasets in which the status of RKIP was experimentally reversed: knockdown in MCF-7 and overexpression in MDA-MB-231 (**Figure 2A**) (16, 17). Inhibiting RKIP expression in MCF-7 cells influenced fewer genes than its overexpression in MDA-MB-231. Moreover, manipulating RKIP resulted in opposing disruption of the regulators of epithelial to mesenchymal transition (EMT). Knocking down RKIP in MCF7 cells partially downregulated several genes in the negative regulators of EMT gene ontology term (GO:000000) (**Figure 2B**). By contrast, overexpressing the RKIP inMDA-MB-231 upregulated the gene products in the same term (**Figure 2C**). Jointly, reversing RKIP status produced a reversal of the regulators of EMT in the two cell line models. This latest finding prompted us to explore the phenotypic effect of RKIP and its regulatory circuit.

**Figure 3:**
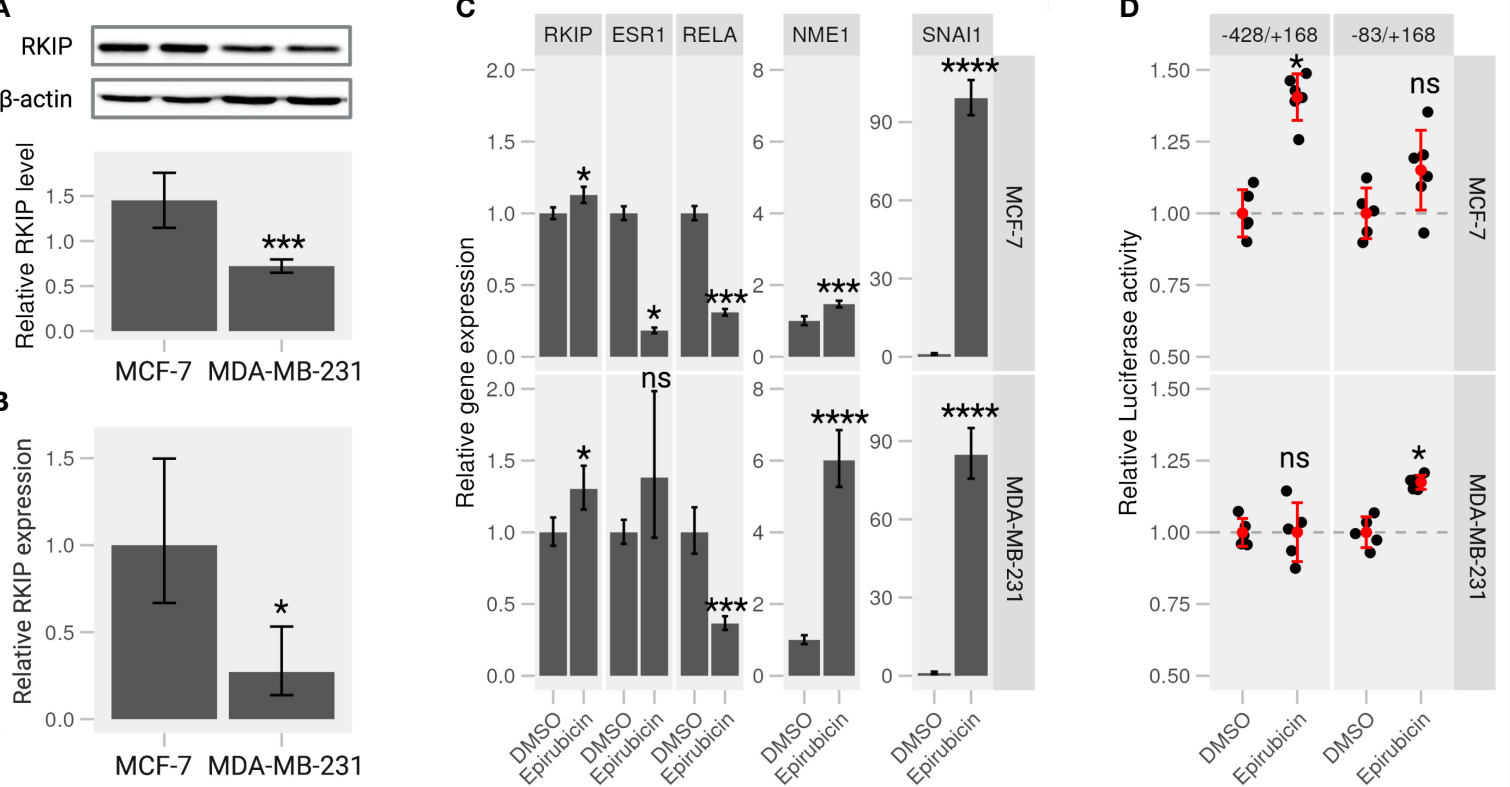
Epirubicin affects RKIP expression and its regulatory pathways on breast cancer cells. **(A)** RKIP expression in MCF-7 and MDA-MB-231 cells. Western blot analysis for RKIP level (upper panel) and the relative RKIP level normalized to beta-actin (bottom panel). Data indicate the mean value ± S.D of three independent experiments.***< 0.005 *p*-value. **(B)** qRT-PCR analysis of RKIP in MCF-7 and MDA-MB-231 cells. The expression of RKIP was quantified in the treated samples relative to the control gene GAPDH. *< 0.05 *p-*value. **(C)** Epirubicin effects on expression of *RKIP* and its potential modulator genes. MCF-7 cells (*upper panel*) or MDA-MB-231 cells (*lower panel*) were treated with Epirubicin (1.5 *μ*M), or the control DMSO for 24 hours. The level of five mRNA (*RKIP*, *RELA*, *ESR1*, extitNME1, and *SNAI1*) was quantified relative to the control *GAPDH*. Data represent the means ± S.D of at least five replicates. *< 0.05, ***< 0.005, ****< 0.001 *p*-values, ns, non-significant. **(D)** Epirubicin effect on the promoter activity of the *RKIP* gene. The relative luciferase activities were determined in MCF-7 or MDA-MB-231 cells transfected with serially deleted constructs (-428/+168 and -83/+168) of the RKIP promoter and treated with Epirubicin or a control DMSO.

We examined the intrinsic RKIP levels in different human cancer cell lines, including breast cancer cells. Indeed, RKIP protein levels varied greatly among the cell lines. Most notably, MCF-7 cells contained higher RKIP compared to MDA-MB-231 (**Figures S2**, **3A**). Additionally, we examined the RKIP mRNA expression by RT-qPCR in the two cell types. MCF-7 expressed more *RKIP* mRNA than MDA-MB-231 cells (**Figure 3B**). This observation is consistent with a published study by S Beach et al. (18). We hypothesized that the RKIP might contribute to the uneven metastatic ability of the two cancer cells. Moreover, manipulating RKIP through chemical substances or its up regulators will be examined.

### 2.3 Targeting RKIP through its transcriptional regulators

In a recent study from our laboratory, we suggested a regulatory circuit including two upstream proteins (SNAI1 and NME1) opposingly regulate the expression of RKIP (19). The study mentioned above and the current results indicate that SNAI1 and NME1 repress and enhance RKIP expression, respectively. We also identified several drugs that activate or repress RKIP in which Epirubicin, one of anthracycline’s antitumor derivatives, among those drugs was found to enhance RKIP expression and perturbs its regulators in the suggested network. We examined the effect of Epirubicin on RKIP expression in the two breast cancer cell lines as suggested in our previous analysis. MCF-7 and MDA-MB-231 cells were treated with the drug for 24h and proceeded for RT-qPCR analysis. We found that Epirubicin significantly induced RKIP expression compared to the DMSO in both cell lines (**Figure 3C**). In MDA-MB-231 cells, we additionally found increased RKIP levels in a dose- and time-dependent manner in response to Epirubicin treatment (**Figures S3B, D**, respectively). Moreover, the influence of these drugs extends to the expression of suggested RKIP upstream regulators. We found that Epirubicin induced the expression of *RKIP* through activating *NME1* instead of through *SNAI1*(**Figure 3C**). The result is consistent across both cell lines and the previous study.

To further examine the effect of Epirubicin on the transcriptional activity of the *RKIP* gene, we constructed several luciferase reporters using serial deletion of the *RKIP* promoter region (**Figure S4A** - *upper panel*). The two regions -83/+168 and -428/+168 produced the highest luminescence signal and were transfected to MCF-7 and MDA-MB-231 cells to measure the *RKIP* promoter activity upon drug treatments (**Figures S4A, B**). Compared to control cells treated with DMSO, Epirubicin was found to enhance the luminescence signal on MCF-7 cells at -428 to +168 construct (*long*), and on MDA-MB-231 cells at -83 to +168 construct (*short*) (**Figure 3D**). This observation indicates that the compound is capable of increasing the expression or transcriptional activity of the *RKIP* gene. To sum up, we confirmed our predictions regarding the effect of Epirubicin on RKIP expression. Moreover, we demonstrated that these effects might occur through one or both of the upstream transcriptional regulators (SNAI1 and NME1).

### 2.4 Identifying the upstream regulators of RKIP

**Figure 4:**
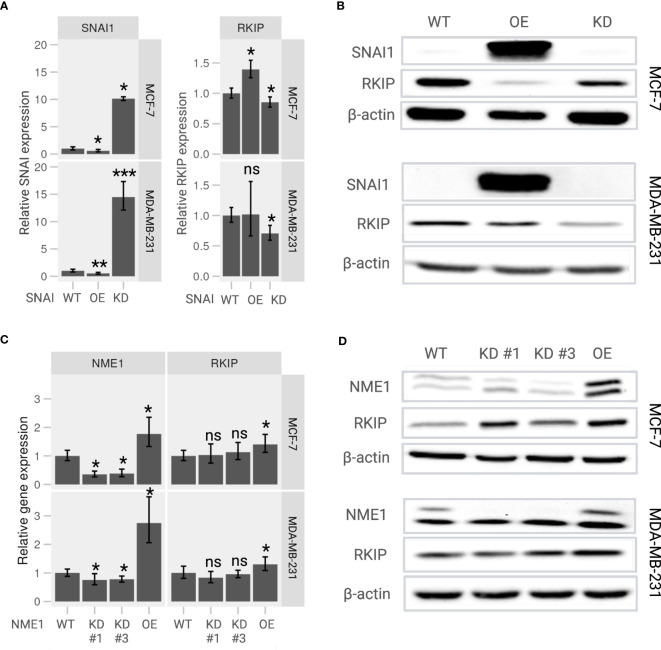
Expression of RKIP in response to modulation of its upstream regulators in MCF-7 and MDA-MB-231 cells. **(A)** Relative *RKIP* expression and **(B)** RKIP protein level in response to SNAI1 knockdown (KD) or overexpression (OE) and compared to the wild-type (WT) MCF-7 (*upper panel*) and MDA-MB-231 (*lower panel*) cells. **(C)** Relative *RKIP* expression and **(D)** RKIP protein level in response to NME1 knockdown (KD) or overexpression (OE) and compared to the wild-type (WT) MCF-7 (*upper panel*) and MDA-MB-231 (*lower panel*) cells. KD #1 and KD #3 are two target-specific shRNA designed to knock down *NME1* expression. The relative mRNA expression in RT-qPCR was normalized to GAPDH. Data represent the means ± S.D of six replicates. *< 0.05; ** < 0.01; *** < 0.005 *p*-values, ns, non-significant.

To further examine the RKIP regulators, we generated knockdown (KD) and overexpression (OE) SNAI1 and NME1 on MCF-7 and MDA-MB-231 cells. As expected, the relative expression of SNAI1 in KD cells was significantly lower and considerably higher in OE cells than wildtype (WT) cells on both the two cell lines (**Figure 4A**, *left panel*). We applied the same experimental design for NME1, and obtained successful OE cells with two KD cells (**Figure 4C**, *left panel*). The observed expression pattern confirmed a successful modification of the two regulators, the consequences of which we explored in terms of changes in the transcriptional activity of the *RKIP* gene.

Overexpressing SNAI1 significantly repressed *RKIP* mRNA compared to WT cells (**Figure 4A**, *right panel*). We observed a similar regulation pattern at the protein level where the RKIP level was inhibited in SNAI OE cells (**Figure 4B**). The effect was observed in both cell lines. These observations are consistent with the work reporting that SNAI1 suppresses RKIP expression (18). In addition, inhibiting SNAI1 enhanced RKIP expression in MCF-7 cells but did not produce a significant effect in MDA-MB-231 cells. Likewise, modifying NME1 expression selectively altered the expression of RKIP at both mRNA and protein levels. Overexpressing NME1 significantly elevated *RKIP* mRNA (**Figure 4C**) and protein levels (**Figure 4D**). RKIP did not change when the same gene was knocked down. Together, these findings support our previously suggested regulatory pathway of RKIP through SNAI1, and NME1 (19).

### 2.5 Overexpressing RKIP inhibits migration of breast cancer cells

**Figure 5:**
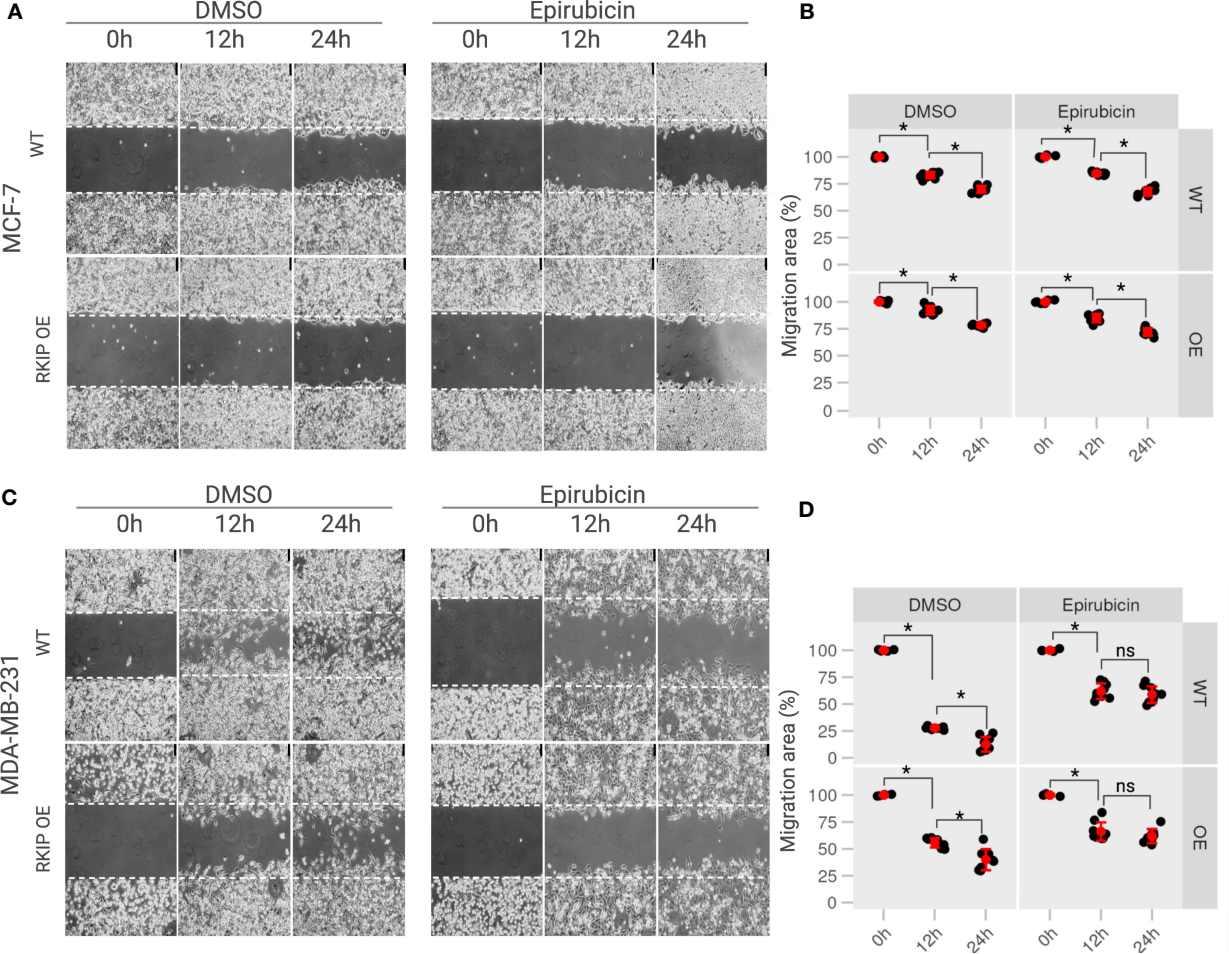
The effects of RKIP expression on the migratory activity of breast cancer cells with Epirubicin. **(A)** MCF-7 cells & **(C)** MDA-MB-231 cells in RKIP wild-type (WT) and RKIP overexpression (OE) were treated with Epirubicin (1.5 *μ*M) and DMSO at 0h, 12h, 24h and used for the wound-healing assay. Cell migration was captured using bright-field microscopy. Bar, 75 *μ*M. **(B, D)** Percentage of migration area (%) from wound healing assays from MCF-7 and MDA-MB-231, respectively quantified from **(A, C)**. Migration area was measured using ImageJ software and graphically represented at 0 h, 12 h, 24 h. The migration area at 0h of conditions was set to 100%. *< 0.05 *p* value, ns, non-significant.

To explore the role of RKIP in breast cancer metastasis *in vitro*, generated transient RKIP overexpression on MCF-7 and MDA-MB-231 cell lines. The overexpression of RKIP on the two cell lines was successfully confirmed by Western blot (**Figure S5**). RKIP is well known by its capability of inhibiting cells metastasis. To confirm that effect on our model, migration ability of MCF-7 and MDA-MB-231 cells with overexpressed RKIP were examined with the scratch assay. As shown on **Figure 5** (*left panel*), compared to WT cells, RKIP overexpression significantly decreased cell migration of MDA-MB-231 cells. MDA-MB-231 cells showed a robust migratory capacity covering 50% of the wound area after 12 h and about95% of that after 24 h in the control group. But the metastatic ability of the cells was inhibited when RKIP is overexpressed, the OE cells reached only 50% at 12 h and cover 60 70% after 24 h. The effect was not observed clearly on MCF-7 cells due to the cell’s low migration ability. The results confirmed that RKIP plays a crucial role in inhibiting breast cancer cells metastasis.

Moreover, we examine the effect of Epirubicin on the migration ability of the two cell lines in RKIP overexpression and RKIP WT using a scratch assay. Epirubicin treatment slightly reduced MCF-7 cell migration at 12h compared to the control on **Figures 5A, B** (*right panel*). Meanwhile it had a significant effect on metastatic MDA-MB-231 cells in which less than 50% of the migration area was covered after 12 h and 24 h following the treatment (**Figures 5C, D**, *left panel*). Similar results were shown in **Figure S1**. Moreover, we observed a similar result on RKIP OE cells where Epirubicin inhibited the migration ability of both cell lines compared to the control groups. Interestingly, we found that Epirubicin mimics over-expressing RKIP effect, especially in MDA-MB-231, in which we found the cell migration was inhibited through either RKIP overexpression or Epirubicin treatment. The differing intrinsic RKIP levels in MCF-7 and MDA-MB-231 could explain their uneven metastatic ability and behavior upon RKIP OE and Epirubicin treatment.

## 3 Discussion

**Figure 6:**
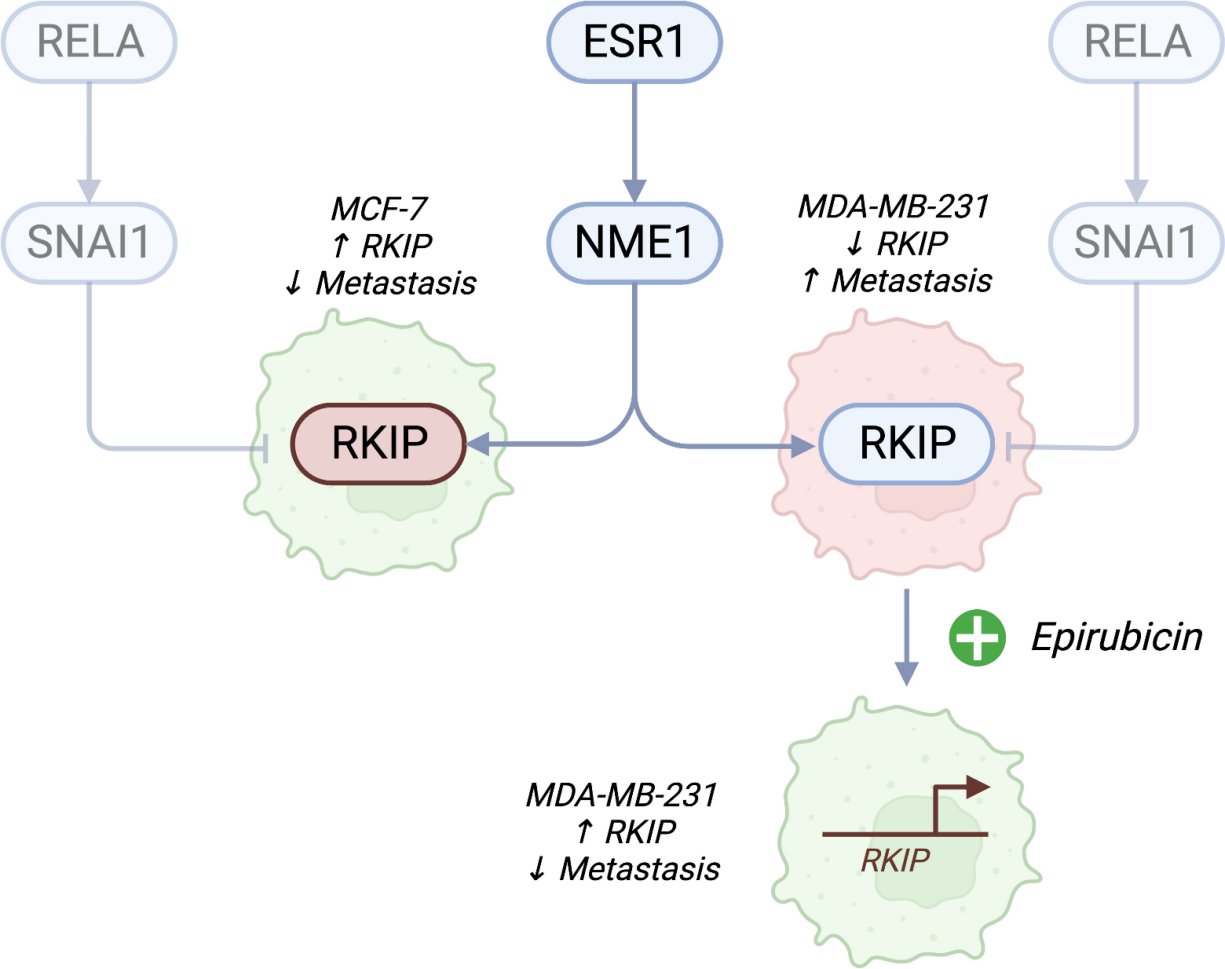
A proposed model of RKIP expression and its regulators on cell migratory activity. Estrogen receptor (ESR1)-negative cancer cells are highly metastatic. In addition to the known effects of losing ER receptors, we propose a connection to RKIP. Cell lines with high RKIP (MCF-7) have low metastatic potential, while cells with low RKIP (MDA-MB-231) are highly metastatic. Pharmacologically activating RKIP (by Epirubicin treatment) increases the expression of the gene and lowers cell migratory abilities. We also propose that the drug promotes the expression of the gene through NME1, which binds to the promoter of RKIP and increases its transcription. Conversely, inhibiting SNAI1 could promote the expression of RKIP. Created with Biorender.com.

In this article, we utilized two breast cancer cells to highlight the role of RKIP as an antimetastatic gene. The expression of RKIP correlates with the metastatic capacity of breast cancer models, among other types of cancer (7, 8). RKIP is also considered a diagnostic biomarker associated with metastasis and poor prognosis (20, 21). MCF-7 cell line has functional estrogen (ER) and EGF receptors and depends on these receptors for growth (13, 22). By contrast, the MDA-MB-231 is a triple-negative breast cancer (TNBC) cell line which does not express the estrogen receptor and grows independently of highly invasive and metastatic human breast cancer (14, 23). RKIP loss has long been recognized to associate in various cancers and it is often absent in highly metastatic aggressive tumors (24–28). Zhang and colleagues (2008) found that loss of RKIP expression was significantly correlated with a lack of ER and progesterone receptor expression (29). A study by Li et al. reported that RKIP was more highly expressed in the original tumors than the metastatic tumors (30). Additionally, a correlation between the expression of RKIP and pRKIP and metastatic ability in melanoma cells was noted in a study of Cardile et al. eCardile2013RafMelanomas. They showed that metastatic melanoma presented downregulation of RKIP and phosphorylated RKIP, while low RKIP and high-phosphorylated RKIP expression may be indicative of non-metastatic melanoma. In this study, we hypothesized that the RKIP could account for the uneven metastatic ability of the two cell types. **Figure 6** shows a diagram of the suggested model.

RKIP was initially suggested as an anti-apoptotic gene that disrupts survival signaling cascades, such as the Raf/MEK/ERK kinase cascades, G protein-coupled receptors (GPCRs), and the NF-kB pathway (31–33). Treating cells with genotoxic drugs may induce the interaction of RKIP with this pathway’s components, subsequently inhibiting cell proliferation, metastasis, and leading to apoptosis induction (34). 4-Shogaol, a phytochemical extracted from red ginger, was found to significantly increase RKIP levels in breast cancer cells, and have a potent effect on reducing tumor growth in transplanted mice (35). Others found that DNA-damaging drugs up-regulate RKIP expression in DU145 prostate cancer cells, and induction of RKIP was not the consequence of apoptosis (7). Moreover, Bonavida et al. showed that Nitric oxide (NO) interferes with the dysregulated NF- κ B/YY1/Snail regulatory circuitry to upregulate RKIP, leading to tumor chemo-immuno-sensitization and suppression of EMT and metastasis (36).

Studies in cell culture and animal models have shown a role for RKIP in acting as an endogenous suppressor of tumor cell survival, proliferation, and metastasis (6, 37). Indeed, further *in-vitro* and *in-vivo* studies showed that exogenous expression of RKIP is sufficient to reduce invasion and metastasis of cancer cells (38, 39). Mice expressing low amounts of RKIP were found to develop tumors earlier and were more resistant to treatment (37). This finding suggests that loss of RKIP increases metastasis in the TRAMP mouse; however, loss of RKIP alone is insufficient to promote metastasis.

In addition to interfering with cell signaling to reduce growth and survival, RKIP inhibits autophagy to the same effect. Several groups have suggested a link between RKIP and key components of autophagy with downstream consequences in terms of cell growth and senescence (40). Work from our laboratory showed that RKIP inhibits autophagy by activating the AKT and MTORC1 or binding to LC3 through an LIR motif (41). In another report, we found that RKIP interacts with EMT and autophagy as part of the same functional unit in developing prostate cancer (42). These reports indicate a promising role of RKIP as a therapeutic target for cancer, either through inhibiting autophagy or its effect on cell growth. Identifying how RKIP is lost in some tumors would be the first step toward developing effective RKIP activators.

Ideally, restoring RKIP expression in all cancer cells *in-vivo* would be the targeting strategy; however, this remains a challenge. Two possible ways by which RKIP expression is inhibited are deletion and reduced transcription of the gene. Recent studies investigated the first possibility and found that a reduced copy number of the RKIP gene does not explain the variation in its abundance across different types of cancer tissues (43, 44). Indeed, our analysis supports this claim. In breast cancer, metastatic tumors were more likely to have the RKIP gene deleted than primary tissues. However, the deletion events happen in only a minority of samples. By contrast, several transcription factors and small RNAs were found to target RKIP in cancer cells selectively (45). We proposed MTDH as a novel factor that controls the RKIP transcription, which is essential for cancer progression (46). We and others suggested SNAI1 as another transcription repressor of RKIP (41, 47). In a recent study, we suggested that RKIP is on the receiving end of two important regulatory mechanisms. One involves RELA (transcription factor p65) and SNAI1, which were previously reported to inhibit RKIP. The other involves the estrogen receptor (ESR1), which induces RKIP through the kinase NME1 (19). We used this same model to predict which cancer drugs target RKIP to produce the desired antimetastatic action.

The model mentioned above was derived in the specific context of breast cancer, but the observed responses to drug treatments were consistent in other cell lines (19). We experimentally validated some predicted regulatory links and drug effects in the breast cancer cell line MCF-7. Here, we examined ranges of targeted genotoxic drugs used as chemotherapy. These included three activators (Epirubicin, Methotrexate, or Vorinostat) and three repressors (Cisplatin, Imatinib, or Sorafenib). Moreover, we specifically showed that the drugs activate or repress RKIP transcriptional activity. We focused on Epirubicin as a promising activator of RKIP. Epirubicin promoted the expression of RKIP through the transcription factor NME1 in both MCF7 and MDA-MB-231. Overexpressing RKIP in the triple-negative breast cancer cell (MDA-MB-231) reduced its migratory pattern. Our previous work placed the estrogen receptor (ESR1) upstream of RKIP regulators. We speculate here that the estrogen stats may relate to RKIP levels and the metastatic ability of breast cancer cells. However, this relation would have to be verified in future works. Moreover, we found that Epirubicin treatment mimicked the RKIP inhibitory role on MDA-MB-231 cells.

The current study was limited to cell models of breast cancer. While valuable at the initial stage of studying novel therapeutic targets and agents, these results need to be replicated *in-vivo*. Furthermore, we established a strong link between the transcription factors NME1 and SNAI1, and RKIP. These factors may be linked to the estrogen receptor ESR1, making it more likely to be relevant to the more aggressive form of breast cancer. However, we did not investigate this link experimentally. Additionally, our analysis of the changes in the cell models in response to RKIP manipulation was limited to morphology and migratory patterns. Other phenotypic changes should be expected and investigated. Finally, these findings should be investigated in other breast cancer models and beyond to test whether they generalize to other cancer types.

## 4 Materials and methods

### 4.1 Gene expression data sets of breast cancer human tissue and cell lines

Two datasets of breast cancer human tissue were obtained from (cbioportal.com): metastatic breast cancer project (MBC; N = 237; primary and metastatic samples) and molecular taxonomy of breast cancer international consortium (METABRIC; N = 2509; primary samples) (11, 12, 48). Processed copy number alterations, receptor status, and gene expression were used in the analysis. Two publically available gene expression datasets of RKIP knockdown in MCF-7 (GSE53668) and overexpression in MDA-MB-231 (GSE128983) were acquired from gene expression omnibus (GEO) (16, 17, 49). Differential expression analysis was performed using limma to compare the knockdown or overexpression to the wild-type cells (50). Enrichment of the gene ontology term (GO:0001837, negative regulation of EMT) was tested in the same comparison using fgsea (51).

### 4.2 Reagents and drugs

Reagents and drugs utilized in this study were purchased as follows: RPMI-1640 media (11875-119), fetal bovine serum (FBS; 16000-044), Epirubicin hydrochloride (CAS 56390-09-1), Vorinostat/SAHA (CAS 149647-78-9), Methotrexate hydrate (CAS 133073-73-1), Cisplatin (CAS 15663-27-1), Sorafenib (CAS 284461-73-0) from Sigma-Aldrich (St. Louis, MO, USA); Imatinib (CAS 220127-57-1) from STEMCELL Technologies Inc. (Vancouver, BC, Canada); Trizol reagent (15596026), PureLink Genomic DNA Mini Kit from Invitrogen (Carlsbad, CA, USA); DNase I Solution (1 unit/ μ L), RNase-free (89836), and RevertAid First Strand cDNA Synthesis Kit from Thermo Scientific (Waltham, MA, USA); amfiSure qGreen Q-PCR Master Mix(2X), Without ROX (Q5600-005) from GenDEPOT (Katy, TX, USA). pGL4.20[luc2/Puro] Vector and pRL-SV40 were purchased from Promega (Madison, WI, USA). Lipofectamine 3000 (11668-500) and G418 (10131-035) were purchased from Gibco and Life Technologies (Carlsbad, CA, USA). M2 lysis buffer (85111), protease inhibitor cocktails (78441), and Enhanced ChemiLuminescence (ECL) detection system (34080) were purchased from Thermo Scientific (Waltham, MA, USA). Antibodies used in the study were as follows: PEBP1/RKIP (sc-28837) from Santa Cruz Biotechnology (Dallas, TX, USA). NME1/NDKA (3345), Snail (C15D3) from Cell Signaling Technology. Secondary antibodies against rabbit (STAR208P) or mouse (STAR117P) were purchased from Bio-Rad (Hercules, CA, USA).

### 4.3 Cell culture, cell transfection, and drugs treatment

MCF-7 and MDA-MB-231 breast cancer cells were cultured in RPMI-1640 supplemented with 10% Fetal Bovine Serum (GIBCO) and 100 μ g/ml streptomycin and incubated in a 37°C humidified atmosphere containing 5% CO _2_. Ectopic expression of target genes (*NME1*, *SNAI1*, *RKIP*) in cells was achieved by transiently transfected with selected plasmids using Lipofectamine 2000 (Invitrogen, CA, USA). Cells were transfected for 16 hours after plating in 6-well plated, and then were used in further assays or RNA/protein extraction. For drug treatment, cells ( $3\times 10^{5}$ cells) were plated in a 6-well plate and further incubated for 24 hours. After that, cells were treated with Epirubicin at 1.5 *μ*M concentration or with DMSO as a control treatment and were collected for assays after 24 hours.

### 4.4 Western blot analysis

Protein extraction, SDS-PAGE, and immunoblotting were performed as previously described (46). Western blot imaging was visualized using the iBright CL1000 Imaging System by Thermo Fisher Scientific. Protein intensity was quantified using the NIHImageJ program (version 1.49). The presented data are the mean ( ± S.D) of at least three independent replicates.

### 4.5 RNA extraction and RT-qPCR

Total RNAs were extracted from cell cultures using Trizol reagent according to the manufacturer’s manual. Prior to cDNA synthesis, RNA samples were treated with DNase I solution to remove trace amounts of DNA. RNA was used as templates for reverse transcriptase to generate First-strand cDNA using Thermo Scientific RevertAid First Strand cDNA Synthesis Kit. The first-strand cDNA synthesis products were used directly in qPCR using the amfiSure qGreen Q-PCR Master Mix kit. Primers of target genes used in the assay are listed in **Table 1**. Gene expression was quantified in the treated samples relative to the control gene GAPDH and the control condition of the DMSO treatment. $\text{ΔΔ}C_{t}$ model was applied using the pcr R package (52). The relative expression of the target genes was compared in each treatment to the control DMSO-treated cells using a student t -test. p-values < 0.05 were considered significant. Experiments were performed in five or more replicates.

**Table 1:** RT-qPCR primers.

Gene	Forward primer	Reverse Primer
ESR1	5’-TGGAGTCTGGTCCTGTGAGG-3’	5’-GGTCTTTTCGTATCCCACCTTTC-3’
SNAI1	5’-CCAGTGCCTCGACCACTATG-3’	5’-CTGCTGGAAGGTAAACTCTGG-3’
RELA	5’-CCTATAGAAGAGCAGCGTGGG-3’	5’-AGATCTTGAGCTCGGCAGTG-3’
NME1	5’-ACTAAGTCAGCCTGGTGTGC-3’	5’-CGCCTTGAAAGACGATCCCT-3’
RKIP	5’-GTCACACTTTAGCGGCCTGT-3’	5’-CTCTCCGATTATGTGGGCTC-3’
GAPDH	5’-TGCACCACCAACTGCTTAGC-3’	5’-GGCATGGACTGTGGTCATGAG-3’

### 4.6 Wound healing assay

Wound-healing assays were used to assess the cell migration ability of MCF-7 and MDA-MB-231 cells *in vitro*. $2\times 10^{5}$ cells/ml of cell suspension were cultured into Culture-Insert 2 Well (#81176, Ibidi, Grafelfing, Germany) and incubated for 24 h to full confluence. The gaps were created by removing the insert, and the cells were monitored for different time points: 0 h, 12 h, 24 h with either Epirubicin (1.5 *μ*M) or DMSO. Images were captured using an Olympus light microscope (Olympus Corporation, Tokyo, Japan). The migration area (%) was measured using ImageJ software, and the migration area at 0h was set to 100%.

### 4.7 Construction of plasmids and luciferase assay

Total genomic DNA was extracted from cell cultures using PureLink Genomic DNA Mini Kit according to the manufacturer’s manual. Different regions of RKIP promoters were amplified by PCR using the total genomic DNA as a template with designed primers (**Table 2**). Constructs were subcloned into the pGL4.20[luc2/Puro] Vector encodes the luciferase reporter gene luc2. All plasmid constructs were verified by restriction enzyme digestion and DNA sequencing (Cosmo Genetech, Korea). To perform luciferase assay, MCF-7 cells were plated at a density of 2×10^5^ cells/well in 96-well plates. The following day, cells were transfected at a ratio of 10:1 of the promoter-luciferase constructs with pRL-SV40 (Promega, Madison, WI, USA) vector expressing Renilla luciferase using lipofectamine 3000. The luciferase activity was measured by the Dual-Glo Luciferase assay system described in the manufacturer’s manual (Promega). The ratio of Firefly/Renilla luminescence in each well was computed. Values were normalized to the ratio obtained from a control well. The presented data are the mean ( ± S.D) of at least six independent experiments.

**Table 2:** Luciferase primers.

No.	Primer	Sequence
1	+1/+168 Forward	5’-GATTCTCGAGATGCCGGTGGA-3’
2	-48/+168 Forward	5’-GATTCTCGAGTGCTCTCGCGT-3’
3	-83/+168 Forward	5’-GATTCTCGAGGTGACGTGG-3’
4	-428/+168 Forward	5’-GATTCTCGAGTTTGGCCTCCCAAAGT-3’
5	-806/+168 Forward	5’-GATTCTCGAGTGTAGTCACAGCTAC-3’
6	+168 Reverse	5’-GATCAGATCTTGACATGCAGCGGGT-3’

### 4.8 Software environment and reproducibility

This analysis was performed in R and using Bioconductor packages (53, 54). The software environment was packaged and distributed as a Docker image (https://hub.docker.com/r/bcmslab/antimetastatic). The code to run the analysis and reproduce the figures and tables in this manuscript is available as open-source (GPL-3) (https://github.com/BCMSLab/rkip_metastasis). Figures were created with Biorender.com.

## Data availability statement

The data analyzed in the article are publicly available on https://www.cbioportal.org and the gene expression omnibus under the following IDs (GSE53668 and GSE128983) or links (https://www.ncbi.nlm.nih.gov/geo/query/acc.cgi?acc=GSE53668 and https://www.ncbi.nlm.nih.gov/geo/query/acc.cgi?acc=GSE128983).

## Author contributions

TL conducted the experiments, analyzed the results, and wrote the manuscript. MA obtained and analyzed the gene expression and copy number alterations data and wrote the manuscript. JH, MB, TP, JY, WK, RM, DL, and D-HK edited the manuscript and supported experiments. HK provided financial support and edited the manuscript. DK provided financial support, supervised the study, and contributed to writing the manuscript. All authors contributed to the article and approved the submitted version.
