# Supplementary material for: Manipulating RKIP reverses the metastatic potential of breast cancer cells
Source: Front Oncol. 2023 Jul 4;13:1189350. doi: 10.3389/fonc.2023.1189350 (PMC10352845; doi:10.3389/fonc.2023.1189350)
Supplement: Supplementary file 1 [file DataSheet_1.pdf]

Supplementary Material

SUPPLEMENTARY FIGURES

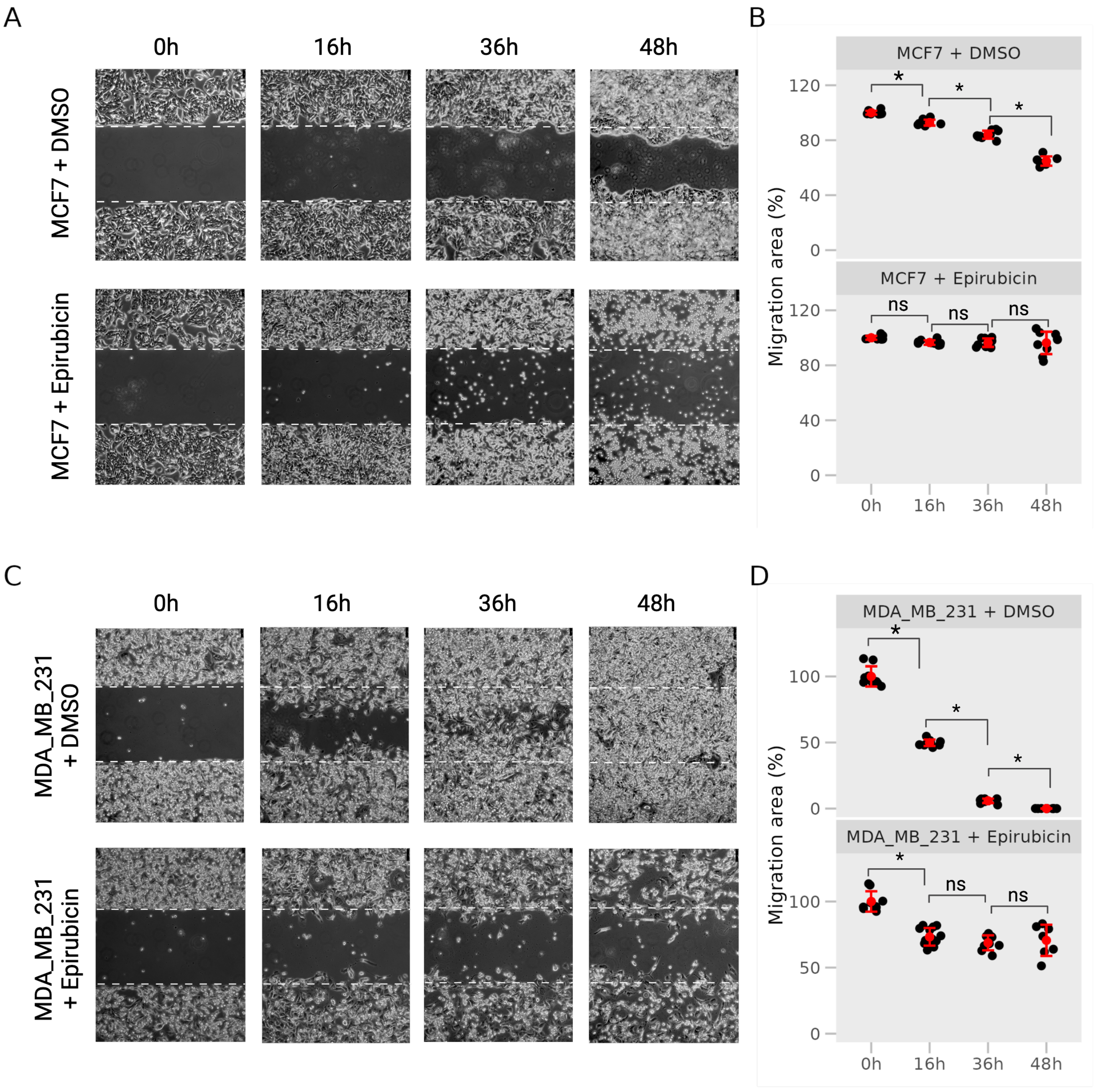

Figure S1. Effect of Epirubicin treatment on the migration ability of MCF-7 and MDA-MB-231 breast cancer cells. Cells, MCF-7 **(A & B)**and MDA-MB-231 **(C & D)** were treated with Epirubicin (1.5 μM) or DMSO in a wound-healing assay. Cell migration was captured using bright-field microscopy. Migration area (%) was captured at 0h, 16h, 36h, and 48h under bright field microscopy and measured using ImageJ software. Bar, 100 μM. At time 0h, the scratch area was set to 100%. $* < 0.05$ p-value.

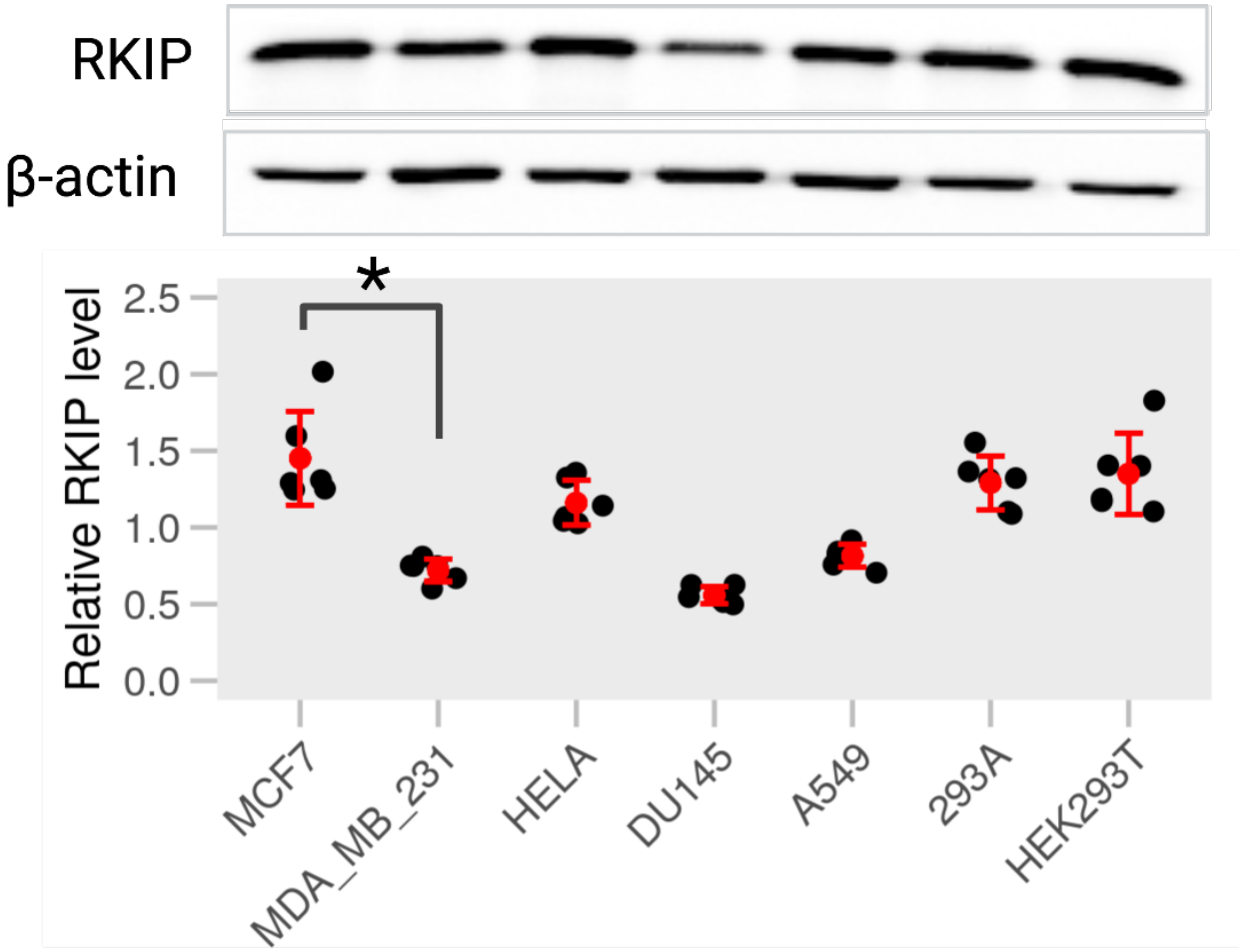

Figure S2. Protein level of RKIP in different cancer cells using western blot (upper panel) and the relative RKIP level normalized to β-actin (bottom panel). Normalized data indicate the mean $\pm$ S.D of at least four independent experiments. $* < 0.05$ p-value. .

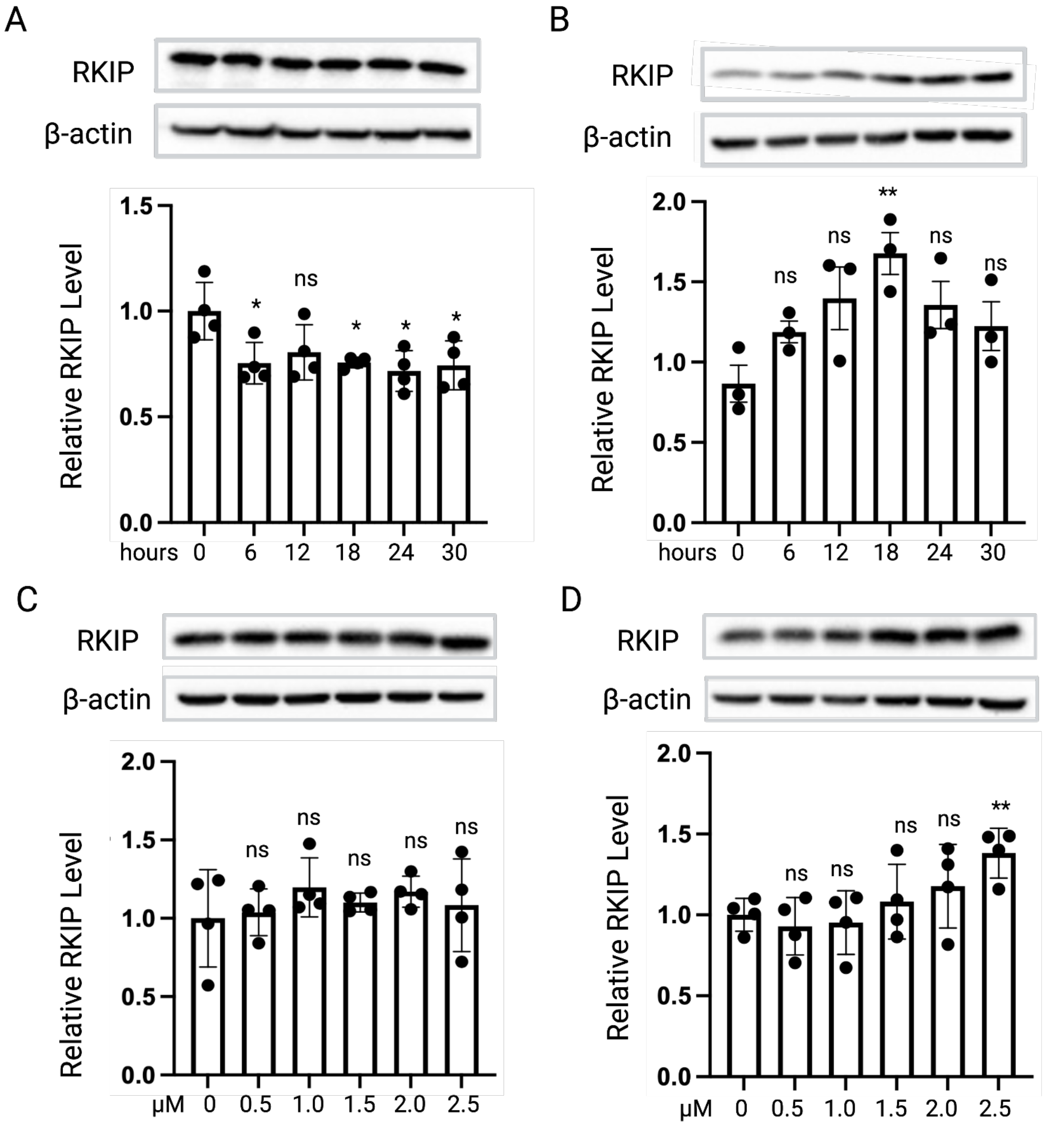

Figure S3. RKIP level in breast cancer cells in response to treatment of Epirubicin. RKIP levels on Epirubicine treatment in a time-dependent manner on (A) MCF-7 cells and (B) MDA-MB-231 cells. RKIP levels on Epirubicine treatment in a dose-dependent manner on (C) MCF-7 cells and (D) MDA-MB-231 cells. For treatment in the time-dependent manner, the cells were treated with Epirubicin (1.5μM), and were harvested after different time points. For treatment in the dose-dependent manner, the cells were treated with different concentrations of Epirubicin for 24 hours. RKIP levels were normalized to β-actin. Data represent the mean ($\pm$ S.D) of three independent experiments. An unpaired t-test was used to test the difference between the group averages. $* < 0.05$, ns: no significance.

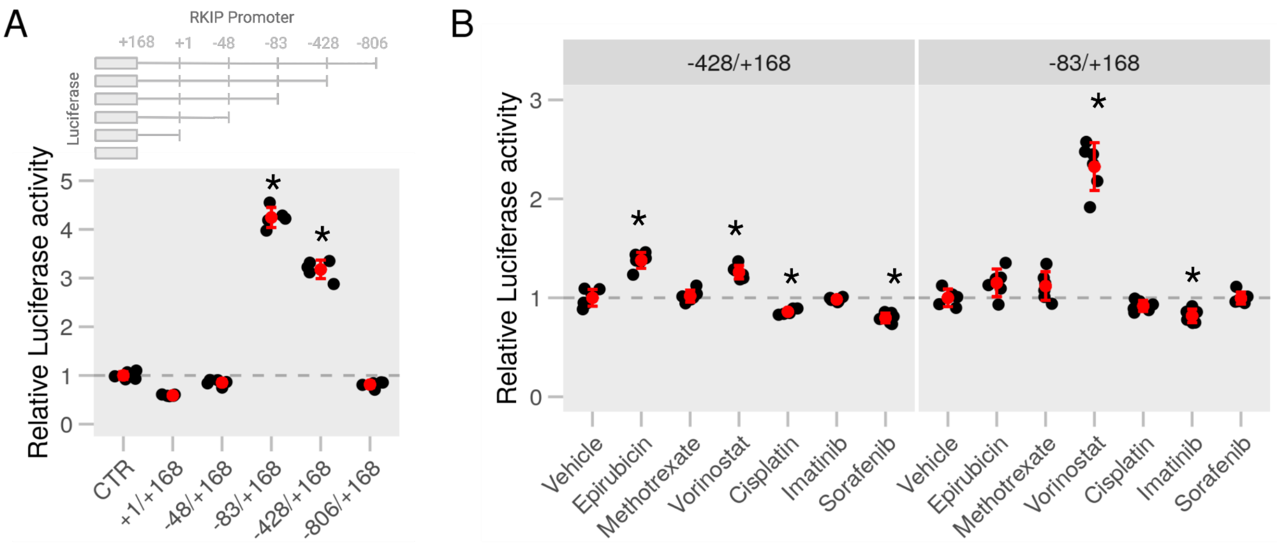

Figure S4. Effects of drugs on RKIP promoter activity of MCF-7 cells. **(A)** A schematic illustration of the constructs for the luciferase RKIP promoter assay (*upper panel*). The serially deleted RKIP promoter DNA fragments (-806/+168, -428/+168, -83/+169, -48/+168, and +/+168) were inserted into the pGL4.20 luciferase vector. (*lower panel*). The relative luciferase activities were determined in MCF-7 cells transfected with the serially deleted constructs of the RKIP promoter. **(B)** RKIP promoter activity upon drug treatments. MCF-7 cells were transfected with *RKIP* promoter region -83/+168 or -428/+168 construct and were treated with three activators (Epirubicin, Methotrexate, or Vorinostat), three repressors (Cisplatin, Imatinib, or Sorafenib), or the control DMSO for 24 hours at 1.5 μM dose. Data represent the means $\pm$ S.D of the relative intensity change from three independent experiments. * $<$ 0.05 p-value.

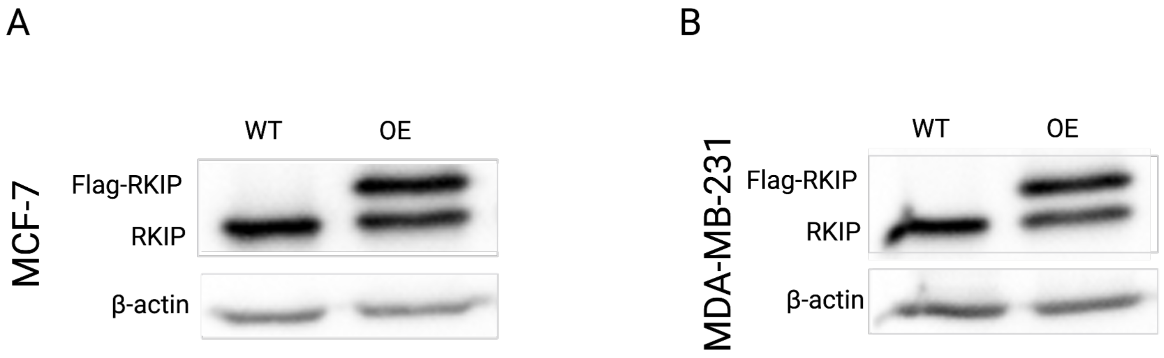

Figure S5. RKIP levels in WT, overexpression (OE) in A) MCF7 and B) MDA-MB-231 cells. The cells at 70% of confluent were transiently transfected with Flag-RKIP constructs for 16 hours. To ensure that all cells undergo the same transfection process and environmental conditions, the WT cells were transfected with the same transfection reaction as the OE cells, but without the specific gene-modifying plasmid. The intensity of bands was quantified using ImageJ. RKIP protein levels were normalized to β-actin. Data represent the mean ($\pm$ S.D) of at least three independent experiments. An unpaired t-test was used to test the difference between the group averages. * $<$ 0.05, ns: no significance.
